# Advanced Microarrays as Heterogeneous Force‐Remodeling Coordinator to Orchestrate Nuclear Configuration and Force‐Sensing Mechanotransduction in Stem Cells

**DOI:** 10.1002/advs.202416482

**Published:** 2025-02-14

**Authors:** Nana Wang, Yan Hou, Lili Lin, Shihui Xu, Kyubae Lee, Yingjun Yang, Yazhou Chen, Yachun Li, Xiuhui Wang, Yongtao Wang, Tao Chen

**Affiliations:** ^1^ Department of Orthopedic Surgery The First Affiliated Hospital of Zhengzhou University Zhengzhou 450052 China; ^2^ Department of Pediatrics Shanghai General Hospital Shanghai Jiao Tong University Shanghai 200080 China; ^3^ School of Medicine Shanghai University Shanghai 200444 China; ^4^ Department of Biomedical Materials Konyang University Daejeon 35365 Republic of Korea; ^5^ Materials Institute of Atomic and Molecular Science Shaanxi University of Science and Technology Xi'an 710021 China; ^6^ Henan Institute of Advanced Technology Zhengzhou University Zhengzhou 450003 China; ^7^ Institute of Translational Medicine Shanghai University Shanghai 200444 China

**Keywords:** advanced microarrays, force‐sensing mechanotransduction, heterogeneous focal adhesion, mechanical remodeling, nuclear configuration

## Abstract

Integrin and focal adhesion can regulate cytoskeleton distribution to govern actin‐related force remodeling and play an important role in nuclear configuration and force‐sensing mechanotransduction of stem cells. However, further exploration of the interaction between actinin complex and myosin, kinetics, and molecular mechanism of cytoskeleton structures to nucleate within the engineered stem cells is vague. An extensive comprehension of cell morphogenesis, force remodeling, and nuclear force‐sensing mechanotransduction is essential to reveal the basic physical principles of cytoskeleton polymerization and force‐related signaling delivery. Advanced microarrays are designed to determine heterogeneous cell morphology and cell adhesion behaviors in stem cells. The heterogeneity from the engineered microarrays is transferred into nuclei to regulate nuclear configuration and force‐sensing mechanotransduction by the evaluation of Lamins, YAP, and BrdU expression. Tuning the activation of adhesion proteins and cytoskeleton nucleators to adjust heterogeneous cell mechanics may be the underlying mechanism to change nuclear force‐sensing configuration in response to its physiological mechanotransduction in microarrayed stem cells.

## Graphical abstract

Engineered microarrays are prepared by the photolithography and further decorated via fibronectin protein to determine the morphogenesis of stem cells. Heterogeneous integrin expression and focal adhesion formation are regulated in microarrayed cells. Polar remodeling of cytoskeleton nanomechanics happens in the heterogeneous cells. Nuclear configuration circulation is explained through force‐sensing mechanotransduction by the evaluation of Lamins, YAP, and BrdU analysis.

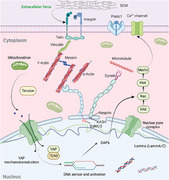

## 1 Introduction

The mechanical properties of cells, including cell hardness, membrane tension and viscosity, and skeleton stress, can not only change the physiological conditions such as cell adhesion, spreading, growth, proliferation, migration, and aging but also play an important role in the evolution and development of various biological diseases.^[^ ^1^ ^]^ An in‐depth study on the mechanical properties of cells is expected to provide a basis for developing new technologies for disease diagnosis and treatment based on mechanical principles.^[^ ^2^ ^]^ At the early stage, the research on cell mechanics may mainly focus on the macroscopic aspects of mechanical force, biology, and medicine, especially for hemodynamics (white blood cells and red blood cells), soft and hard tissue mechanics (cartilage and bone).^[^ ^3^ ^]^ In recent years, many researchers have carried out a lot of work on cell mechanics at the micro or nano level.^[^ ^4^ ^]^ For instance, micropatterns and nanopillars can adjust cell adhesion behaviors and cell cytoskeleton distribution to determine cell nanomechanics and movement.^[^ ^5^ ^]^ The exploration of bioengineering and cell mechanics as a new interdisciplinary subject is still in the rising stage, and the further theoretical knowledge system still needs to be developed and improved in the mechanism of cell mechanics.^[^ ^6^ ^]^ Therefore, it is of great significance for the investigation of micro‐cell mechanics to disclose the molecular mechanism of cell nanomechanics and its potential in disease diagnosis and therapy.

The influence of single‐cell geometry topology on heterogenous nanomechanics in cells is also important to seek out the underlying reason for force remodeling.^[^ ^7^ ^]^ Some studies have reported that the geometric morphology of single cells (microns and nanostructures) has an impact on cell functions.^[^ ^8^ ^]^ Cells with large spreading areas and adhesion areas can improve the nanomechanical strength of cells and promote bone differentiation and regeneration.^[^ ^9^ ^]^ These biophysical cues in heterogeneity will rearrange the formation of adhesion spots and cytoskeleton structure, thus changing the nanomechanical behaviors of the engineered cells.^[^ ^10^ ^]^ Cell microenvironments, such as chemical composition, hardness, and viscosity of matrix, can induce cell nanomechanics and monitor the delivery ability of exogenous genes by the regulation of nuclear heterogeneous mechanics.^[^ ^11^ ^]^ The gene delivery and expression in nuclei can manipulate the process of gene knockout or overexpression, protein modification, and cell reprogramming from decline to prosperity, and strengthen the active exploration of cell heterogeneous mechanics in nuclear force‐sensing mechanotransduction.^[^ ^12^ ^]^ In this study, we used the engineered microcircles to manipulate cell morphogenesis on heterogeneous microarrays. Heterogeneous integrin expression and focal adhesion formation were regulated in engineered cells to affect cytoskeleton distribution and force remodeling. Cytoskeleton‐related nanomechanics could explain nuclear configuration circulation by motor molecule evaluation and nuclear force‐sensing mechanotransduction in heterogeneous cells. The study will give the valuable perspectives for the understanding of cell heterogeneity, cytoskeletal mechanics, and nuclear force‐sensing mechanotransduction.

## 2 Results

### 2.1 Conceptualization and Characters of Engineered Microarrays

**Figure 1:**
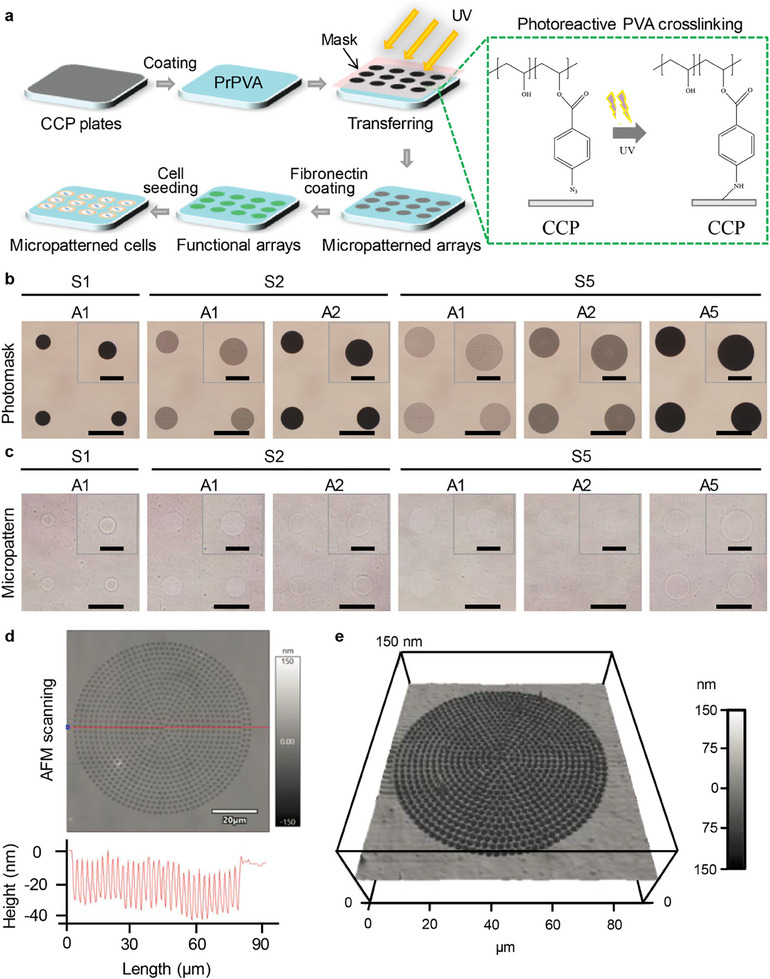
Conceptualization and characterization of the engineered microarrays. a) Preparing illustration of the heterogeneous microarrays by photoreactive PVA cross‐linking process. b) Representative pictures of the designed photomasks. Scale bar: 100 µm. Inserts are the partial enlargement of a single microcircle. Scale bar: 50 µm. c) Representative pictures of prepared microarrays. Scale bar: 100 µm. Inserts are the partial enlargement of a single microcircle. Scale bar: 50 µm. d) AFM scanning and cross‐section in S5A2 microarrays. e) 3D view of S5A2 microarrays by AFM analysis.

Biofunctional engineered microarrays were conceptualized and fabricated by intelligent poly(vinyl alcohol) (PVA) modification via photolithography as a heterogeneous coordinator of cell nanomechanics to determine nuclear force‐sensing mechanotransduction and remodeling. Intelligent PVA solution was synthesized by grafting a photoreactive azide group (4‐azidobenzoic acid) into PVA chains to construct a photo‐triggered PVA polymerization system. The heterogeneous microarrays with various adhesion and spreading areas were prepared by the UV‐crosslinking PVA technology on cell culture plates (CCP) (**Figure** **1**a). The successful design for heterogeneous independence of spreading and adhesion area was also created by laser irradiation on silicon wafer substrates (Figure 1b). In detail, the microarrays contained bulk and dark microcircles (40, 60, and 80 µm in diameter) or the same diameter microcircles with black microdots in diameter of 2 µm. Adhesion area (A) was defined to be 1256 µm^2^ (A1), 2826 µm^2^ (A2), and 5024 µm^2^ (A5), while spreading area (S) was 1256 µm^2^ (S1), 2826 µm^2^ (S2), and 5024 µm^2^ (S5), respectively. 2‐µm‐in‐diameter microdots were schemed to tune the heterogeneous independence of spreading and adhesion areas. The desired microarrays were completely consistent with the designs of six‐type microarrays on the photomask. Afterward, the well‐designed photomask was adopted to construct the polystyrene (PS) microarrays on PVA‐modified CCP plates. The formation of heterogeneous PVA/PS microarrays that were surrounded by PVA areas was confirmed by the observation with optical microscopy. The as‐prepared PVA/PS microarrays were the same as the design of the photomask (Figure 1c), showing good controllability and stability for microarray preparation.

Further, the integration of each microarray was also analyzed by the scanning pictures of AFM. A representative AFM scanning picture of the S5A2 microarray was presented to prove the precise adjustment of heterogeneous microarrays (Figure 1d). In addition, the 3D view was also shown by AFM scanning (Figure 1e). The measured results indicated that the diameters of micropatterns (40.5, 60.9–61.7, and 81.0–82.0 µm) and microdots (2.0–2.3 µm), spreading area (1287.2, 2915.6–2991.9, 5205.5–5285.9 µm^2^) and adhesion area (1287.2–1433.3, 2728.1–2991.9, 5149.1 µm^2^) well agreed with the pre‐designed ones on microarrays and the thickness (39.7–42.6 nm) of PS/PVA microarrays (Table S1, Supporting Information). Therefore, the AFM outcomes demonstrated that these engineered microarrays could be accurately controlled by adjusting PrPVA concentration, content and grafting ratio.

### 2.2 Engineered Cell Morphogenesis on Heterogeneous Microarrays

**Figure 2:**
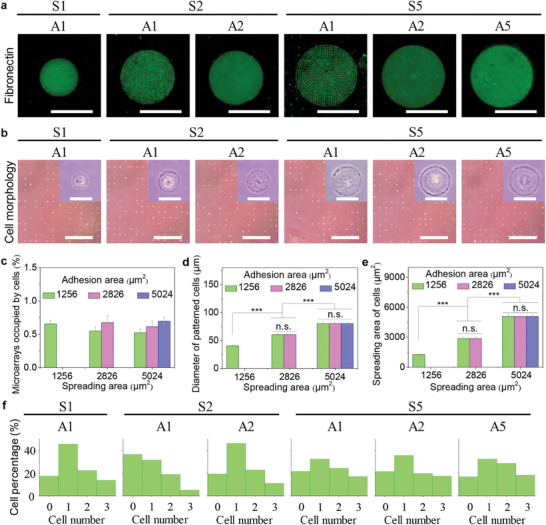
Engineered cell morphology on heterogeneous microarrays. a) Representative pictures of fibronectin‐coated microarrays. Scale bar: 50 µm. b) Cell morphology of the engineered hMSCs on heterogeneous microarrays. Scale bar: 1000 µm. Inserts are the partial enlargement of single cells. Scale bar: 50 µm. c) Percentage of microarrays occupied by cells. d) Diameter of patterned cells. e) Spreading area of patterned cells. The data present mean ± SD, n = 5, n.s., no significance; ^***^ *p* < 0.001. f) Relationship of cell number and cell percentage in each microarray.

The engineered and topological structures can play the decisive role in modulating cell morphogenesis.^[^ ^13^ ^]^ The engineered microarrays were used to regulate heterogeneous cell adhesion and spreading areas in the same patterned plates. To improve the adhesion ability of human bone marrow‐derived mesenchymal stem cells (hMSCs) on heterogeneous microarrays, fibronectin solution was cast onto the engineered microarrays. Further, the fibronectin (Fn)‐coated microarrays were confirmed by immunostaining and the results showed that fibronectin was only absorbed in the microarrayed regions, but not in non‐patterned regions (**Figure** **2**a). The hMSCs suspension at P4 was dropped onto the engineered microarrays and deposited into each micropattern during 6 h culture. After 24 h incubation on the microarrays, the topological morphology of the engineered hMSCs was regulated by the heterogeneous microarrays to adapt to these circular microarrays (Figure 2b). The cell seeding results presented that the percentage of microarrays occupied by cells was over 50% on each type of microarray (Figure 2c). The diameters of the engineered cells were calculated to be 40.5±1.2, 60.5±1.3, 60.4±1.7, 80.5±1.4, 80.3±0.9, and 80.4±1.3 µm for S1A1, S2A1, S2A2, S5A1, S5A2, and S5A5, respectively (Figure 2d). Meanwhile, the spreading area of the engineered hMSCs was analyzed by ImageJ software and the calculated spreading area (1287.2±74.4, 2875.9±121.9, 2873.3±158.2, 5086.6±179.1, 5076.8±111.2, and 5080.7±159.3 µm^2^ for S1A1, S2A1, S2A2, S5A1, S5A2, and S5A5, respectively) was almost the same with designed ones (Figure 2e). The single cell in each microarray was evaluated by nuclear analysis (Figure S1a, Supporting Information). The percentage of single cells in microarrays was >30–40% (Figure 2f). In addition, the engineered cells were stained by the live/dead double staining method (Figure S1b, Supporting Information), and over 90% of cells were alive, indicating high cell survival ability on the engineered microarrays (Figure S1c, Supporting Information). Collectively, the engineered cell morphogenesis was well adjusted without decreasing cell viability by the heterogeneous microarrays.

### 2.3 Heterogeneous Adhesion Behaviors on Heterogeneous Microarrays

**Figure 3:**
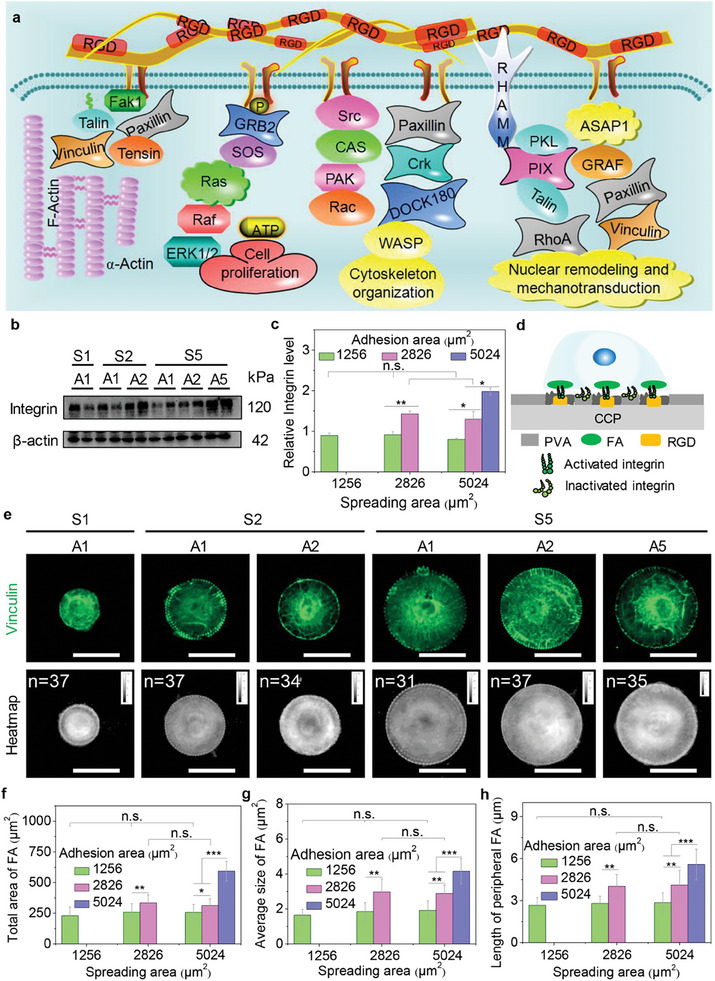
Heterogeneous adhesion protein expressions in the engineered hMSCs. a) Schematic diagram of adhesive molecule clutch assembly to show that adhesion proteins (integrin or cadherin) on the cell membrane are activated by extracellular microenvironment to control FA formation and filamentous actin organization, thus regulating cell functions. b) WB analysis of integrin. c) Relative integrin expression level (n = 4). d) Illustration of activated integrin to induce RGD and FA formation on heterogeneous microarrays. e) Representative pictures of vinculin staining. The heatmap of vinculin is formed by stacking >30 pictures along with the z‐axis into one image. Scale bar: 50 µm. f) Total area of FA in the engineered hMSCs. g) The average size of FA in a single cell. h) Length of peripheral FA. The data present mean ± SD, n = 5, n.s., no significance; ^*^ *p* < 0.05; ^**^ *p* < 0.01; ^***^ *p* < 0.001.

The assembly of molecular clutch has shown that the adhesion proteins (integrin or cadherin) on the cell membrane are instantly activated by extracellular ligands in microenvironment to control focal adhesion formation and filamentous actin organization, thus regulating various cell functions (**Figure** **3**a).^[^ ^14^ ^]^ The engineered hMSCs were cultured on the microarrays and integrin was evaluated by western blot (WB) analysis (Figure 3b). The WB results presented that large cell adhesion area on the microarrays (S2A2, S5A2, and S5A5) could enhance integrin expression level, while small adhesion area (S1A1) may suppress integrin expression even though the cells had a larger spreading area (S2A1 and S5A1) (Figure 3c). Interestingly, integrin expression was improved by increasing cell adhesion area, independently of cell spreading area on the heterogeneous microarrays.

The engineered microarrays could activate integrin by biofunctional fibronectin microdots to regulate focal adhesion (FA) formation (Figure 3d). To prove this hypothesis of integrin activation to induce FA assembly, vinculin, a main component of FA, was stained green and captured by a laser confocal microscope to investigate the FA formation of the engineered hMSCs on the microarrays (Figure 3e). The stained FA results revealed that all cells could produce vinculin on engineered microarrays based on single cell level (Figure S2a, Supporting Information). In addition, the heatmaps of FA‐tracking hMSCs were also analyzed by stacking >30 FA images along with the z‐axis into one heatmap image (Figure S2b, Supporting Information). The heatmap images showed that more vinculin was observed in large adhesion area cells, independently of the cell spreading area. Further, the quantitative vinculin was calculated by the step‐by‐step method by ImageJ software (Figure S2c, Supporting Information) to analyze the FA formation. The total area of FA was enhanced with increasing cell adhesion area from 1256 to 5024 µm^2^, while cell spreading area (1256, 2826, and 5024 µm^2^) did not affect FA formation in 1256 µm^2^ adhesion area cells (Figure 3f). The average size of FA showed similar results with the total area on the engineered microarrays. The cells had the largest average FA size (4.2±0.7 µm^2^) on S5A5 microarrays, but the cells decreased their FA size to 1.6 ± 0.3 µm^2^ on S1A1 microarrays, independently of cell spreading area (Figure 3g). Moreover, the length of peripheral FA was also measured to evaluate the heterogeneous assembly of peripheral FA on the engineered microarrays (Figure 3h). The biased length of peripheral FA exhibited the increasing tendency in the cell adhesion area but was not affected by the cell spreading area. In addition, the expression level of vinculin protein was also detected by WB analysis to disclose the expression level of FA‐related proteins (Figure S3, Supporting Information). The WB results showed that the vinculin expression level presented the positive relationship with the adhesion area, independently of the spreading area. Therefore, heterogeneous adhesion behavior (FA formation) was adjusted by activating integrin and vinculin on heterogeneous microarrays.

### 2.4 Polar Remodeling of Cytoskeleton Nanomechanics on Heterogeneous Microarrays

**Figure 4:**
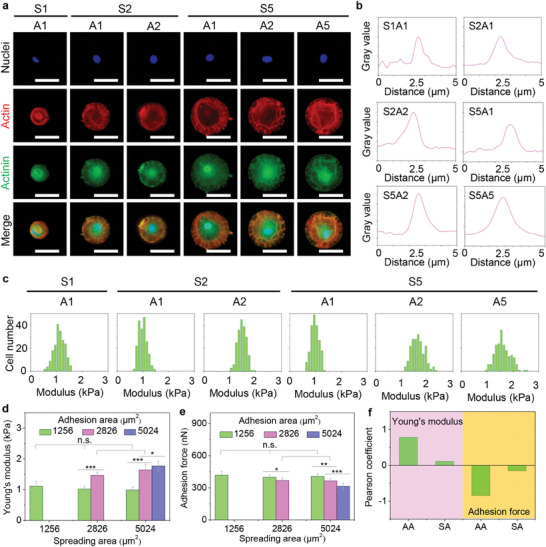
Cytoskeletal remodeling and polar nanomechanics on heterogeneous microarrays. a) Representative pictures of cytoskeleton‐related staining. Nuclei: blue; red: actin; green: actinin. The last row is merged pictures. Scale bar: 50 µm. b) The thickness of cytoskeletal filaments (actinin). c) Relationship of Young's modulus and cell number in each engineered hMSC. d) Average Young's modulus of heterogeneous cells. e) Adhesion force of hMSCs. The data present mean ± SD, n = 5, n.s., no significance; ^*^ *p* < 0.05; ^**^ *p* < 0.01; ^***^ *p* < 0.001. f) Pearson coefficient analysis between cell adhesion behaviors and Young's modulus or adhesion force.

Heterogeneous adhesion behaviors, including integrin expression and FA formation, could manage cytoskeleton assembly and distribution to guide cytoskeletal nanomechanics on the engineered microarrays. Cell cytoskeleton fibers (actin and actinin) were investigated to reveal the regulation of heterogeneous microarrays on the polar remodeling of cytoskeleton nanomechanics. First, actin was detected to disclose the cytoskeletal organization on the engineered microarrays (Figure S4a, Supporting Information). The actin results showed that the thickness of actin was significantly increased with cell adhesion area but not affected by cell spreading area (Figure S4b, Supporting Information). Actin and actinin were co‐stained to observe the positioning situation of force‐sensing proteins (**Figure** **4**a). More actinin fibers were formed and co‐located with actin in large adhesion area hMSCs, while this phenomenon is not found in small adhesion area even the cells had large cell spreading area. Afterward, we performed that actinin fibers were quantitatively calculated to distinguish the thickness of actinin fibers by ImageJ analysis (Figure S4c, Supporting Information). The results presented that the thickness of actinin fibers was enhanced with increasing cell adhesion area, independently of cell spreading area (Figure 4b). Specially, S5A5 cells were calculated to have the largest thickness of actinin fibers, up to 3.0 µm per fiber. These staining results indicated that heterogeneous actin and actinin fibers were constructed to direct cytoskeleton remodeling on engineered microarrays.

Cellular nanomechanics may be closely related to force‐sensing cytoskeleton structures to regulate cell functions.^[^ ^15^ ^]^ The mechanical properties were explored to unveil polar cytoskeleton nanomechanics in the microarrayed hMSCs. Young's modulus of the engineered hMSCs was measured by AFM to manifest the polar stiffness of cells on heterogeneous microarrays (Figure S5a, Supporting Information). The cellular stiffness showed an increasing tendency with enhancing cell adhesion area and further cell number having high Young's modulus was also lifted in large adhesion area cells (Figure 4c). The quantitative cell stiffness presented that Young's modulus was increased to 1.85 kPa in S5A5 cells, while the modulus was 1.13 kPa in S5A1 cells, independently of cell spreading area (Figure 4d). Cell adhesion force was also investigated to reveal the adhesion force between the cell membrane and the AFM cantilever when returning the AFM probe. The measurement results indicated that cell adhesion force was reduced in the engineered cells with well‐organized cytoskeleton structures and the lowest adhesion force (370 nm) was observed in S5A5 cells, while the cell spreading area had little influence on cell adhesion force (Figure 4e). The relationship between cell adhesion behaviors and heterogeneous nanomechanics was further inspected by Pearson's coefficient analysis to evaluate the action mechanism of cytoskeleton‐induced heterogeneity on polar remodeling of cellular nanomechanics on the microarrays (Figure 4f). The cell stiffness showed a positive correlation with the cell adhesion area, while the adhesion area had a negative influence on cell adhesion force. Interestingly, both cell stiffness and adhesion force were not affected by cell spreading area. In addition, the cytoskeleton actin fibers were inhibited by cytochalasin D to prevent actin filaments from polymerizing on the engineered microarrays. The mechanical results showed that Young's modulus (Figure S5b, Supporting Information) and cell adhesion force (Figure S5c, Supporting Information) exhibited the same results in all microarrayed cells. Similarly, there was no relationship between cell adhesion and nanomechanics in the engineered cells (Figure S5d, Supporting Information). Therefore, the cytoskeleton results demonstrated that adhesion‐mediated cytoskeleton heterogeneity could promote polar remodeling of cellular nanomechanics on the engineered microarrays.

### 2.5 Nuclear Configuration Circulation by Motor Molecule Evaluation

**Figure 5:**
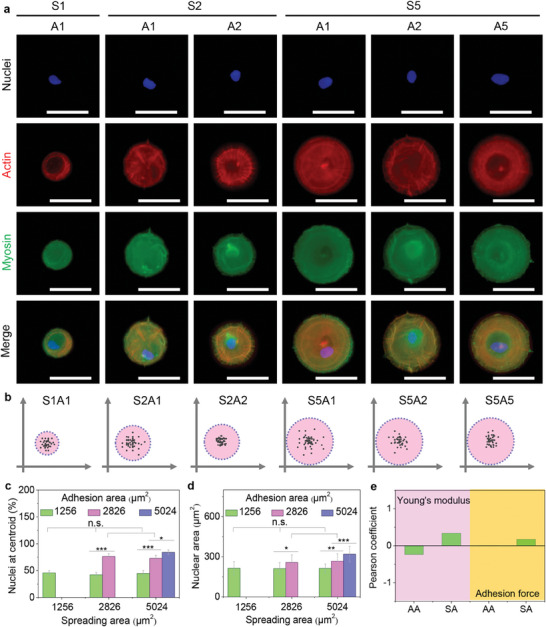
Nuclear configuration alternation by motor molecule evaluation. a) Representative pictures of motor myosin molecules (green). Nuclei: blue; actin: red. The last row is merged pictures. Scale bar: 50 µm. b) Discrete distribution of nuclei on heterogeneous microarrays. c) Percentage of nuclei at centroid in the engineered cells. d) Nuclear area of the heterogeneous cells. The data present mean ± SD, n = 5, n.s., no significance; ^*^ *p* < 0.05; ^**^ *p* < 0.01; ^***^ *p* < 0.001. e) Pearson coefficient analysis between cell adhesion behaviors and Young's modulus or adhesion force after myosin disturbance.

The engineered cytoskeleton nanomechanics played a crucial role in regulating intracellular motor‐related molecule expression. Myosin is one of the main motor proteins to supervise nuclear configuration circulation by force‐sensing cytoskeleton transmission on heterogeneous microarrays.^[^ ^16^ ^]^ Myosin motor proteins were evaluated by immunofluorescent staining to monitor the influence of cell nanomechanics on the assembly and distribution of motor molecules in the engineered cells (**Figure** **5**a). Myosin fibers were well formed and co‐located with actin filaments in S5A5 cells, while the fiber‐like network was dissolved in small adhesion area cells even the cells displayed large spreading area (S5A1) on the microarrays. The expression difference of myosin motor proteins could affect nuclear position information on heterogeneous microarrays (Figure 5b). More nuclei preferred the central regions of the engineered microcircles in S5A5 cells, indicating the consistent result with myosin expression. Moreover, the nuclei at the centroid were also amounted to disclose the nuclear centrality by cytoskeleton‐induced nanomechanics and force‐sensing motor molecule expression (Figure 5c). S5A5 cells presented a higher percentage of nuclei at the centroid compared to S5A2 and S5A1. Nuclear force‐sensing configuration could change the nuclear area due to the biased cytoskeleton mechanics and heterogeneous myosin distribution (Figure 5d). The nuclear area was enhanced in S5A5 cells, while the cell spreading area did not adjust the nuclear area even in large spreading area cells. To further certify nuclear force‐sensing configuration circulation by motor‐induced nanomechanics, the myosin was disturbed by blebbistatin to supervise the relevance of motor molecules and cellular nanomechanics on the microarrays. The myosin fibers disappeared by inhibitor treatment in all microarrayed cells (Figure S6a, Supporting Information). Young's modulus (Figure S6b, Supporting Information) and adhesion force (Figure S6c, Supporting Information) also presented the same results. The Pearson's coefficient analysis showed no significant difference in cell adhesion behaviors and cell nanomechanics after myosin disturbance (Figure 5e). The percentage of nuclei at the centroid was found to indicate the conformable tendency with <50% level for the treated cells (Figure S6d, Supporting Information). Overall, the nuclei were adjusted to adopt the centralization of microarrays and nuclear force‐sensing configuration circulation was regulated by cytoskeleton nanomechanics and motor molecule evaluation.

### 2.6 Nuclear Force‐Sensing Mechanotransduction on Heterogeneous Microarrays

**Figure 6:**
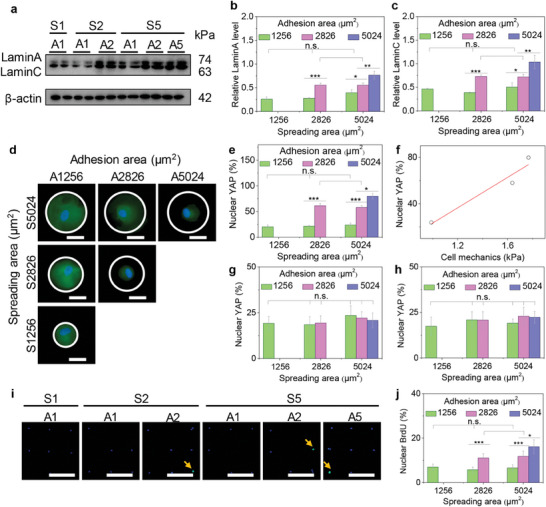
Nuclear force‐sensing mechanotransduction in the heterogeneous hMSCs. a) LaminA/C measurement by WB analysis. b) Related LaminA expression level. c) Related LaminC expression level (n = 4). d) Representative pictures of YAP staining (green). Scale bar: 50 µm. The white circles present cell spreading areas on heterogeneous microarrays. e) Percentage of nuclear YAP. f) Relationship of cell mechanics and nuclear YAP translocation. g) Percentage of nuclear YAP after actin disturbance. h) Percentage of nuclear YAP after myosin disturbance. i) Representative pictures of BrdU staining (green). Nuclei: blue. Scale bar: 200 µm. The yellow arrows point out BrdU‐positive nuclei. j) Percentage of nuclear BrdU on the engineered microarrays. The data present mean ± SD, n = 5, n.s., no significance; ^*^ *p* < 0.05; ^**^ *p* < 0.01; ^***^ *p* < 0.001.

Microarray‐induced heterogeneous cell adhesion behaviors could alter integrin expression and FA formation to regulate polar cytoskeleton nanomechanics, thus affecting nuclear patterning configuration. To further verify the differentiated nuclear activity, nuclear force‐sensing mechanotransduction was also evaluated on heterogeneous microarrays. Nuclear lamins was located between the inner nuclear membrane and chromatin to support nuclear mechanics and participate in nuclear information expression on microarrays. LaminA/C was evaluated to explore the expression level by WB analysis in the engineered cells (**Figure** **6**a). The WB results indicated that the relative expression levels of LaminA (Figure 6b) and LaminC (Figure 6c) were significantly enhanced in S5A5 cells, while small adhesion area did not have almost any influence on the expression levels of the nuclear lamins. After cytoskeleton (actin filaments) polymerization inhibition treatment, nuclear lamins was also detected by WB analysis (Figure S7a, Supporting Information). The WB results displayed that LaminA (Figure S7b, Supporting Information) and LaminC (Figure S7c, Supporting Information) had the homogeneous expression levels in all microarrayed cells. YAP nuclear translocation was explored to reveal nuclear heterogeneous remodeling via force‐sensing mechanotransduction in the engineered hMSCs (Figure 6d; Figure S7d, Supporting Information). The percentage of nuclear YAP presented an increasing tendency with enlarging cell adhesion area, independently of cell spreading area, indicating the similar results with nuclear lamin expression (Figure 6e). Further, YAP nuclear translocation had a positive relationship with cell nanomechanics, which was associated with microarray‐induced adhesion heterogeneity and polar cytoskeleton assembly (Figure 6f). To reveal the role of cytoskeleton‐induced mechanics in YAP nuclear translocation, the cytoskeleton structures of actin filaments and motor proteins of myosin were respectively disturbed by cytochalasin D (Figure 6g) and blebbistatin (Figure 6h), and YAP nuclear translocation had the consistent level in engineered cells. The lamins and YAP results suggested that nuclear force‐sensing remodeling was adjusted by cytoskeleton‐related mechanotransduction.

**Figure 7:**
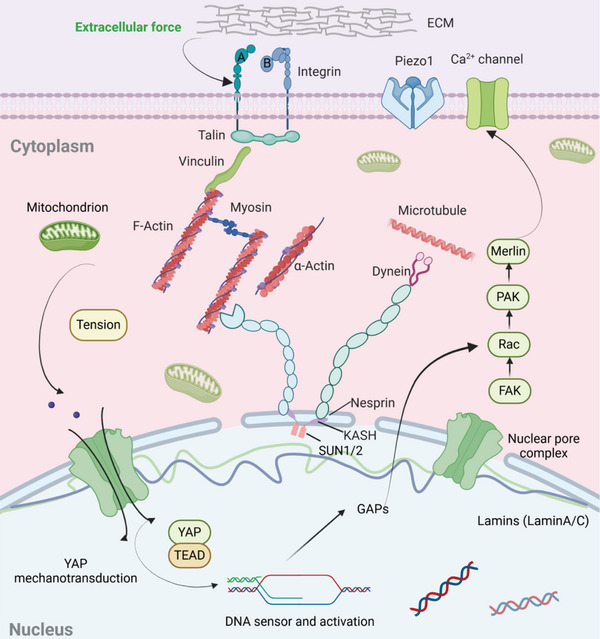
Illustration of extracellular force transmission from heterogeneous microarrays into cells to regulate nuclear mechanotransduction and DNA synthesis. Cell mechanics may be transduced into the cells through integrin induction to activate FAK and tyrosine kinase Src (TK Src). LATS 1/2 kinase and YAP are phosphorylated to inhibit its combination with TEAD and finally regulate proliferation‐related gene expression. The heterogeneous microarray was designed to serve as a coordinator of biased nanomechanics to determine nuclear force‐sensing mechanotransduction and DNA synthesis via lamins and YAP expression.

The nuclear heterogeneous mechanotransduction may regulate nuclear activity by cytoskeleton‐induced nanomechanics.^[^ ^17^ ^]^ DNA synthesis was evaluated by BrdU staining to inspect the connection between nuclear force‐sensing remodeling and nuclear activity (Figure 6i; Figure S7e, Supporting Information). More BrdU‐positive cells were observed in large adhesion area cells (S5A5 cells). The percentage of BrdU‐positive cells was increased with enhancing cell adhesion area, while there was no significant difference in cell spreading cells (Figure 6j), implying that nuclear force‐sensing mechanotransduction induced by heterogeneous nanomechanics promoted DNA synthesis on the engineered microarrays. Collectively, the engineered biofunctional microarrays could serve as the heterogeneous coordinator of cell nanomechanics by the regulation of integrin and FA formation to determine nuclear force‐sensing remodeling and DNA synthesis via nuclear lamin expression and YAP mechanotransduction (**Figure** **7**).

## 3 Discussion

Cell heterogeneity is a common and important phenomenon, and presents the remarkable difference in morphogenesis, functionalization and molecular characteristics to explain the physiological evolution of tissue/organ development and detect the pathological mechanisms of cancers and autoimmune diseases.^[^ ^18^ ^]^ Engineered biomaterials, including 3D scaffolds, mechanical hydrogels and micropatterning have been widely designed to regulate cell‐interface heterogeneity, such as cell source heterogeneity, gene expression heterogeneity and functional heterogeneity.^[^ ^19^ ^]^ Although enormous studies have focused on cell heterogeneity in molecular mechanisms and disease therapy, the influence of heterogeneous cell nanomechanics on nuclear force‐sensing remodeling via mechanotransduction is rarely explored. In this study, the heterogeneous microarrays were prepared to adjust cell nanomechanics by the regulation of heterogeneous integrin‐FA coupling complexes and determine nuclear force‐sensing remodeling via mechanotransduction (Figure 1).

Heterogeneous cell adhesion is the basic element to consider cell‐interface interaction and cell heterogeneity.^[^ ^20^ ^]^ Integrin is the receptor of cell adhesion to serve as the communication bridge between the cells and adhesion surfaces.^[^ ^21^ ^]^ Transmembrane protein and heterodimer protein receptors bind to arginine‐glycine‐aspartic acid (RGD) and can promote cell heterogeneous adhesion.^[^ ^22^ ^]^ FA kinase (FAK) is activated to induce cell adhesion behaviors on heterogeneous surfaces.^[^ ^23^ ^]^ It has been reported that spatial patterning in heterogeneous cell populations is related to clustering and sorting by varying cell‐cell and cell‐interface adhesion.^[^ ^24^ ^]^ Heterogeneous signaling transmission will participate in affecting pathophysiological evolution in energy metabolism and disease development.^[^ ^25^ ^]^ Therefore, the heterogeneous microarrays were designed by the synthesized PVA to regulate the heterogeneous adhesion behaviors of stem cells and induce heterogeneous integrin and FA formation in the engineered cells (Figure 3). The adhesion proteins prefer forming adhesion dots on the microarrays to stimulate the adhesion‐related signaling pathways, including FAK, Rho‐mediated kinase, mitogen‐related protein kinase, and protein kinase C for intracellular adhesion signaling transduction of heterogeneous response.^[^ ^26^ ^]^

Cellular nanomechanics and response have been verified to play a decisive role in cell adhesion and spreading, migration and movement, proliferation and differentiation, embryonic development, tissue formation, and disease development.^[^ ^27^ ^]^ The FA‐actin coupling structures are adjusted by the heterogeneous integrin‐FAK signaling pathway on the cell membrane to activate the intracellular biomechanical signals for the changes of heterogeneous cell nanomechanics.^[^ ^28^ ^]^ Specially, the microarrayed hMSCs could perceive extracellular microenvironment (heterogeneous coordinator) to promote regional heterogeneous adhesion proteins and trigger adhesion‐related downstream signals (GTPase and RhoA).^[^ ^29^ ^]^ Integrins transmit the mechanical signal of material interface transmitted by fibronectin into intracellular FAK, thus prompting paxillin to reorganize and assemble tubulin and actin. Cadherin can directly sense extracellular mechanical signals, which are transferred to β‐catenin and α‐catenin, and the α‐catenin affects cytoskeleton reconstruction.^[^ ^30^ ^]^ The biased integrin and FA formation were regulated by the engineered microarrays to administrate heterogeneous cytoskeleton orientation (actin, actinin, and myosin) and incongruous tension evolution (Figures 4 and 5). The mechanical stimuli can induce Tyrosine kinase activation to phosphorylate the binding site between G3bp2 proteins and Twist 1 proteins, thus dissociating both proteins.^[^ ^31^ ^]^ Twist 1 protein accumulates at the promoter of SNAI2 gene through nuclear translocation to induce the generation of vimentin, inhibit E‐cadherin expression, and eventually promote epithelial‐mesenchymal transition (EMT) by heterogeneous cytoskeleton nanomechanics.^[^ ^32^ ^]^

Extracellular mechanical stimulation is transmitted to the nucleus through the linkers of nucleoskeleton and cytoskeleton complexes (LINC) and the nucleus responds to the mechanical signal through the changes of nuclear membrane proteins, nuclear pore complexes, lamin proteins, and chromatin structures (Figure 6).^[^ ^33^ ^]^ Nuclear force‐sensing deformation, including nuclear membrane stretching, nuclear membrane rupture/repair, DNA damage, and nuclear‐cytoplasmic transport can affect cell migration, division and cell fate.^[^ ^34^ ^]^ Cell mechanics may be transduced into the cells through integrin induction to activate FAK and tyrosine kinase Src (TK Src).^[^ ^35^ ^]^ LATS 1/2 kinase and YAP/TAZ are phosphorylated to inhibit its combination with transcriptional enhanced associate domain (TEAD) and finally regulate proliferation‐related gene expression.^[^ ^36^ ^]^ Together, the engineered microarrays were applied to act as a heterogeneous coordinator of biased nanomechanics to regulate nuclear force‐sensing mechanotransduction and DNA synthesis via lamins and YAP expression (Figure 7). These mechanical results in the microarrayed stem cells may be beneficial for potential applications in stem cell therapy and tissue regeneration.^[^ ^37^ ^]^

## 4 Conclusion

The heterogeneous microarrays were designed as a coordinator of mechanical remodeling to regulate nuclear configuration and force‐sensing mechanotransduction in the engineered hMSCs. The characters of engineered microarrays were measured by AFM and well agreed with the designed parameters. Heterogeneous cell morphology was engineered to distinguish between cell adhesion area and cell spreading area on microarrays. Integrin expression and FA formation showed that the cell adhesion behaviors could be controlled in the heterogeneous hMSCs. Polar remodeling of cytoskeleton‐induced nanomechanics (actin and actinin) was constructed on the microarrays. Heterogeneous cell nanomechanics could determine nuclear configuration circulation by motor molecule (myosin) evaluation. Nuclear force‐sensing mechanotransduction and DNA synthesis were also affected by the regulation of lamins, YAP and BrdU expression. Thus, this study may provide some valuable information for heterogeneous nanomechanics and nuclear force‐sensing mechanotransduction in stem cells.

## 5 Experimental Section

### Design, Production, and Characterization of Engineered Microarrays

The engineered microarrays with adhesion and spreading heterogeneity were fabricated based on cell culture polystyrene (CCP) plates via UV photolithography. In brief, photoreactive poly(vinyl alcohol) (PVA) was modified by grafting the azidophenyl group in 4‐azidobenzoic acid to PVA chains to produce the azidophenyl‐derived photoreactive PVA (PrPVA). The synthesized PrPVA solution was dropped on CCP plates (a square area of 1.5×1.5 cm^2^) to form a thin PVA nanolayer after air drying in the dark. Then, a designed mask including the microarrays with adhesion and spreading heterogeneity was covered on the CCP plates to suffer from 254‐nm UV exposure. The microdots with the diameter of 2 µm were designed on the microarrays to distinguish between heterogeneous adhesion and spreading area. The microarrays were designed to have different adhesion areas and spreading areas. For example, the diameters of microcircles were set as 40, 60, and 80 µm, and the corresponding spreading areas were 1256, 2826, and 5024 µm^2^, thus defined as S1, S2 and S5, respectively. The adhesion areas of 1256, 2826, and 5024 µm^2^ were defined as A1, A2, and A5. After ultrasonic wash, the microarrayed CCP plates were prepared for cell culture. The microarrays were captured by the microscope.

The geometrical topography of engineered microarrays was evaluated through an atomic force microscope (MFP‐3D‐BIO AFM, Asylum Research, USA). The AFM cantilever containing 0.06 N/m spring constant and 12–24 kHz oscillation frequency was used to analyze the microarrays. The cantilever was set on a circular holder and approached the engineered microarrays to measure the geometrical topography. AFM scanning regions were fixed as the square area (90×90 µm^2^) in a water‐contact mode. The characters of microarrays including diameter, height, and thickness were acquired based on three random microcircles.

Prior to cell inoculation on the microarrays, the CCP plates were immersed in 75% ethanol for sterilization. To enhance cell inoculation ability on microarrays, the CCP plates were coated with fibronectin with the concentration of 20 µg mL^−1^ (Sigma–Aldrich, USA). Fibronectin was dissolved in pH = 8.4 NaHCO_3_. The fibronectin‐coated CCP plates were stained by an anti‐fibronectin primary antibody (Santa Cruz Biotechnology, USA) and Alexa Fluor‐488 labeled antibody (Invitrogen, USA). The stained microarrays were captured by a fluorescent microscope.

### Cell Micropatterning and Cell Culture on Microarrays

Human bone marrow‐derived mesenchymal stem cells (hMSCs, passage 2) were bought from Lonza Walkersville Inc in the USA and subcultured to passage 4 (P4) in mesenchymal stem cell growth bulletkit medium (MCSGM, Lonza Group Ltd., Switzerland) in a 5% CO_2_ incubator, based on manufacturer protocol. The cells at P4 with a 1.0×10^5^ cells/mL cell density in cell culture flasks were obtained by trypsin‐EDTA treatment. Before the cells were seeded on the microarrays, fibronectin‐modified CCP plates were put into 6‐well culture plates and supplemented with 3 mL pre‐warmed high glucose Dullecco's modified eagle's medium (DMEM) with 10% fetal bovine serum. A square PDMS framework (1.5 cm × 1.5 cm) was placed on the micropatterned plates to prevent the cell leakage. Subsequently, 0.1 mL cell suspension (≈1×10^4^ cells) was dropped into each PDMS framework to assist cellular adhesion and spreading on the microarrays. Next, the cells were cultured on the microarrays for 6 h to remove the PDMS framework, and the engineered cells were continuously incubated for another 18 h. A phase‐contrast microscope was used to observe the cell morphology and circular structures. The percentage of microarrays occupied by cells, diameter of patterned cells, and spreading area of cells were evaluated by ImageJ software. The percentage of single cells on the engineered microarrays was analyzed by nuclear Hoechst 33258 staining.

### Immunofluorescent Staining of Vinculin

After incubation of microarrayed hMSCs for 24 h, the cells were washed with PBS three times and fixed with 4% paraformaldehyde (PFA) for 10 min. Then, the cells were treated with 1% Triton X‐100 for 10 min and 0.02% Tween‐20 for 30 min, followed by 3 washes. The cells were also blocked with 2% BSA for 30 min. After PBS washing, the cells were immersed in mouse anti‐vinculin antibody (Merck KGaA, Germany) in a warm incubator for 1.5 h. The samples were rinsed with 0.02% Tween‐20 and PBS 3 times. The cells were stained with Alexa Fluor 488 labeled anti‐mouse IgG antibody (Invitrogen, USA) in the dark for 1 h. The nucleus was stained with Hoechst 33258 in the dark for 10 min. The fluorescent pictures were captured by a laser confocal scanning microscope (LCSM, Olympus FV3000, Japan). The vinculin heatmap pictures were obtained by stacking >30 pictures along with z‐axis. The total area of focal adhesion (FA), average size of FA, and length of peripheral FA were analyzed by ImageJ software.

### Immunofluorescent Staining of Cytoskeletal Structures

Cytoskeletal structures including actin, actinin, and myosin were evaluated by immunofluorescent staining. The microarrayed cells were fixed with PFA. For actin and actinin staining, the microarrayed cells were permeabilized with 1% Triton X‐100 for 10 min and blocked with 1% BSA for 30 min, respectively. Then, an aqueous solution of mouse anti‐actinin antibody (Abcam, USA) was dropped onto the blocked cells and incubated at 4°C overnight. After PBS washing, the cells were stained with Alexa Fluor 488 labeled goat anti‐mouse IgG antibody (Invitrogen, USA) for 1 h in the dark. Next, nuclei and actin filaments were stained with Hoechst 33258 for 10 min and Alexa Fluor‐594 phalloidin (Invitrogen, USA) for 20 min in the dark. The thickness of actin fibers was calculated by ImageJ software. For myosin staining, the microarrayed cells were permeabilized by 1% Triton X‐100 for 10 min and blocked by 2% BSA for 30 min, respectively. After PBS washing, myosin was stained with rabbit anti‐myosin IIA antibody (Sigma–Aldrich Co. LLC., USA) and Alexa Fluor 488 anti‐rabbit IgG antibody (Invitrogen, USA). Nuclei and actin filaments were stained with Hoechst 33258 for 10 min and Alexa Fluor‐594 phalloidin for 20 min in the dark. Further, the nuclear location was analyzed to calculate the percentage of nuclei at the centroid and nuclear area by ImageJ software. In addition, after incubation of the microarrayed hMSCs for 24 h, the engineered cells were respectively treated with 0.2 µg mL^−1^ cytochalasin D (Cyto D, Sigma–Aldrich, USA) and 50 µm Blebbistatin (Bleb, Sigma–Aldrich, USA) in DMEM medium for 6 h. Then, the staining was carried out in the same method as the above protocols.

### Nanomechanical Properties of Engineered Cells in Microarrays

An MFP‐3D‐BIO AFM with a point‐contact nanoindentation system was used to characterize nanomechanical properties of the engineered cells on the microarrays. Si_3_N_4_ cantilever with 0.06 N/m nominal spring constant (Novascan, USA) was set onto a circular holder. Before the measurement of cell nanomechanics, the corrected spring constant of the cantilever was calculated by the thermal noise method based on the manufacturer protocol. The indentation parameters (indentation rate: 4 µm s^−1^; trigger force: 2 nN) were selected as a constant. After the cells were incubated for 1 day, they were measured by the fixed nanoindentation system to analyze the nanomechanics of the microarrayed cells, including cell stiffness and adhesion force. In this process, the cells were observed to find the highest‐point location and then touched the 600‐nm‐in‐diameter silica sphere. Each assay was executed within 2 h. Next, PUNIAS software was used to calculate Young's modulus (cell stiffness) and adhesion force from force‐distance curves. The linear fitting option in the Hertz contact model was used to correct baseline tilt by constant Poisson ratio (0.5). The Pearson coefficient between cell nanomechanics and cell adhesion or spreading was also evaluated. Further, actin and myosin structures were also disturbed to investigate the influence of cytoskeletal structures on nanomechanics. The engineered cells were treated with Cyto D and Bleb to disturb the formation of actin and myosin fibers. AFM was used to measure cell nanomechanical properties in the same protocol.

### YAP Expression Evaluation in Microarrayed Cells

After the engineered cells were cultured for 24 h in a warm incubator, they were treated with PFA for 10 min, 1% Triton X‐100 for 10 min, and 2% BSA for 30 min, respectively. The monoclonal mouse anti‐YAP primary antibody (Cell Signaling Technology, USA) was used to incubate the cells at 4 °C overnight and the second antibody (Alexa Fluor 488‐conjugated IgG antibody) was used to stain the cells. The nuclei were stained with Hoechst 33258 in the dark for 10 min. The percentage of YAP nuclear translocation was calculated by ImageJ software. In addition, the engineered cells were treated with Cyto D and Bleb to disturb the formation of actin and myosin fibers. The influence of cytoskeletal structures on nuclear YAP translocation was evaluated.

### Regulation of DNA Synthesis by 5‐Bromo‐2’‐Deoxyuridine (BrdU)

DNA synthesis was analyzed by BrdU (Thermo Fisher, USA) staining. The engineered cells were incubated in an FBS‐free medium for 24 h to starve the engineered cells into the G0 state. Then, the G0‐state cells were seeded on the microarrays for 24 h. Opti‐MEM medium with 1% BrdU‐labeled reagents was used to replace the DMEM growth medium for 24 h. After that, the engineered cells were fixed with 75% ethanol for 30 min, treated with 2 m HCl solution for 30 min, 1% Triton X‐100 for 10 min, and 2% BSA for 30 min, respectively. Finally, the hMSCs were incubated with monoclonal mouse anti‐BrdU primary antibody (Abcam, USA) for 1.5 h and stained with Alexa Fluor 488‐conjugated anti‐mouse IgG antibody (Thermo Fisher, USA) in the dark for 1 h. The nucleus was stained with Hoechst 33258 at room temperature in the dark for 10 min. The percentage of BrdU‐positive nuclei was analyzed through fluorescent pictures.

### Western Blot (WB)

The expression levels of vinculin, integrin, and LaminA/C proteins were detected by WB analysis. Proteins of interest were extracted in engineered cells. Protein lysate was prepared by mixing 10 µL protease inhibitor and 10 ml tissue protein lysate. The engineered cells were treated with protein lysate and put on ice for 20 min. The treated cells were centrifuged at a speed of 12 000 g at 4 °C for 20 min to remove the cell debris and collect the supernatant. BCA Protein Assay Kit was used to determine protein content. After the protein concentration of the test samples was uniformly quantified, 4× loading buffer containing β‐mercaptoethanol was added to proteins and heated at 100°C for 5 min. SDS‐PAGE protein electrophoresis was executed. The prepared protein samples were put into the prepared gel and voltage power was set as 100 V for electrophoresis. The gel‐separated samples were transferred to a PVDF membrane with a constant current of 300 mA and 1 min/KD protein. 5% skimmed milk powder (1% PBST configuration) was used to block the samples at room temperature for 2 h, and washed with 1% PBST 3 times, 5 min/ time. The respective primary and second antibodies for vinculin, integrin, and LaminA/C were used to incubate the samples. The developer was dropped onto the samples to expose vinculin, integrin and LaminA/C proteins in an exposure apparatus. The WB results were calculated to analyze expression levels of vinculin, integrin and LaminA/C proteins by ImageJ software.

### Statistical Analysis

The data present average ± standard deviation (SD). The difference in the quantitative results was analyzed by one‐way ANOVAs with a Tukey's post‐test for multiple variables and confidence results were carried out for the data in KyPlot 5.0. The difference was considered when *p* < 0.05. n.s., no significance; ^*^ *p* < 0.05; ^**^ *p* < 0.01; ^***^ *p* < 0.001.

## Conflict of Interest

The authors declare no conflict of interest.

## Supporting information



Supporting Information

## Data Availability

The data that support the findings of this study are available from the corresponding author upon reasonable request.
