# Supplementary material for: Advanced Microarrays as Heterogeneous Force‐Remodeling Coordinator to Orchestrate Nuclear Configuration and Force‐Sensing Mechanotransduction in Stem Cells
Source: Adv Sci (Weinh). 2025 Feb 14;12(14):2416482. doi: 10.1002/advs.202416482 (PMC11984837; doi:10.1002/advs.202416482)
Supplement: Supplementary file 1 — Supporting Information [file ADVS-12-2416482-s001.docx]

**Supporting Information**

**Advanced Microarrays as Heterogeneous Force-Remodeling Coordinator to Orchestrate Nuclear Configuration and Force-Sensing Mechanotransduction in Stem Cells**

Nana Wang^1,2#^, Yan Hou^3,#^, Lili Lin^2,#^, Shihui Xu^3^, Kyubae Lee^4^, Yingjun Yang^5^, Yazhou Chen^6^, Yachun Li^2,^*, Xiuhui Wang^7,^*, Yongtao Wang^3,^*, Tao Chen^1,^*

^1^ Department of Orthopedic Surgery, The First Affiliated Hospital of Zhengzhou University, Zhengzhou 450052, China

^2^ Department of Pediatrics, Shanghai General Hospital, Shanghai Jiao Tong University, Shanghai 200080, China

^3^ School of Medicine, Shanghai University, Shanghai 200444, China

^4^ Department of Biomedical Materials, Konyang University, Daejeon 35365, Republic of Korea

^5^ Materials Institute of Atomic and Molecular Science, Shaanxi University of Science and Technology, Xi’an 710021, China

^6^ Henan Institute of Advanced Technology, Zhengzhou University, Zhengzhou 450003, China

^7^ Institute of Translational Medicine, Shanghai University, Shanghai 200444, China

^#^These authors contributed equally to this work.

*Correspondence author:

Prof. Yachun Li, E-mail: [yachunli@126.com](mailto:yachunli@126.com)

Prof. Xiuhui Wang, E-mail: [blackrabbit@shu.edu.cn](mailto:blackrabbit@shu.edu.cn)

Dr. Yongtao Wang, E-mail: [yongtao_wang@shu.edu.cn](mailto:yongtao_wang@shu.edu.cn)

Dr. Tao Chen, E-mail: [zzuchentao@yahoo.com](mailto:zzuchentao@yahoo.com)

**Table S1.** The characters of heterogeneous microarrays include diameter, spreading area, adhesion area, size of microdots, and PVA thickness. The data present mean ± SD, n = 3.

| Pattern types | S1A1 | S2A1 | S2A2 | S5A1 | S5A2 | S5A5 |
| --- | --- | --- | --- | --- | --- | --- |
| Diameter (μm) | 40.5±0.9 | 60.9±1.4 | 61.7±1.4 | 81.4±1.8 | 82.0±0.7 | 81.0±1.2 |
| Spreading area (μm^2^) | 1287.2±44.8 | 2915.6±112.3 | 2991.9±72.4 | 5205.5±192.1 | 5285.9±71.5 | 5149.1±124.6 |
| Adhesion area (μm^2^) | 1287.2±44.8 | 1318.5±281.0 | 2991.9±72.4 | 1433.3±214.8 | 2728.1±530.2 | 5149.1±124.6 |
| Microdot size (μm) | non | 2.2±0.2 | non | 2.0±0.4 | 2.1±0.5 | non |
| PVA thickness (nm) | 41.3±2.3 | 40.7±1.8 | 41.4±0.9 | 39.7±2.0 | 41.0±1.4 | 42.6±1.9 |

**
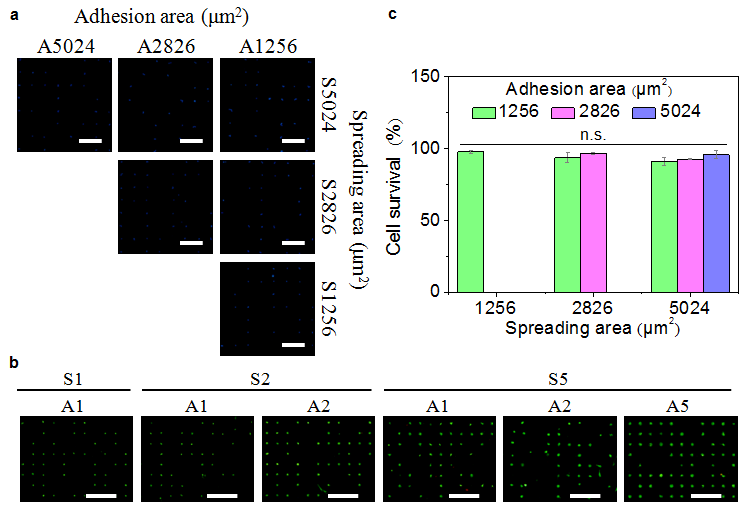
**

**Figure S1.** (a) Representative pictures of nuclear staining (blue). Scale bar: 500 μm. (b) Representative pictures of live/dead double staining method. Scale bar: 500 μm. (c) Percentage of cell survival on heterogeneous microarrays. The data present mean ± SD, n = 5, n.s., no significance.

*
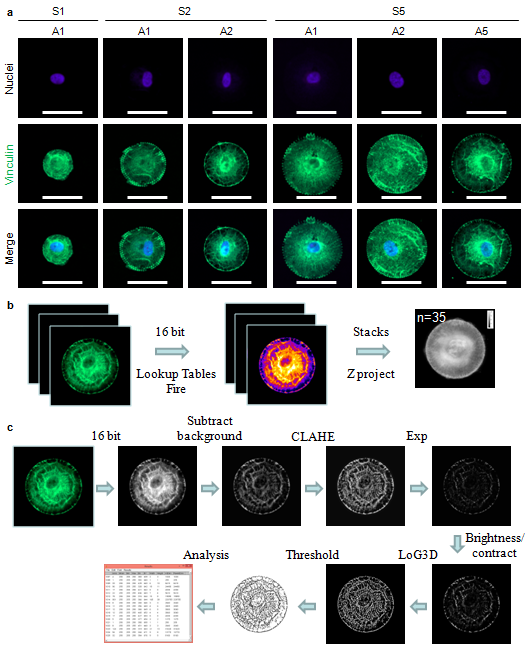
*

**Figure S2.** (a) Representative pictures of FA analysis by vinculin staining (green). Nuclei: blue. The last row is merged pictures. Scale bar: 50 μm. (b) Flow chart on how to make the heatmap pictures of vinculin by z-axis stacking. (c) A step-by-step method to analyze the total area and average size of focal adhesion.


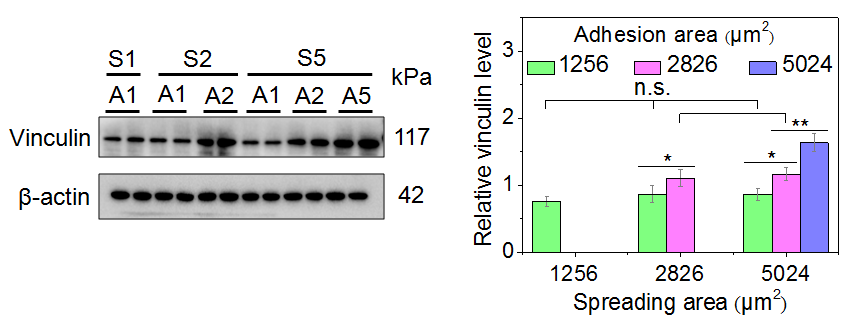


**Figure S3.** WB analysis to detect the expression level of vinculin protein in microarrayed cells. The data present mean ± SD, n = 4, n.s., no significance.

*
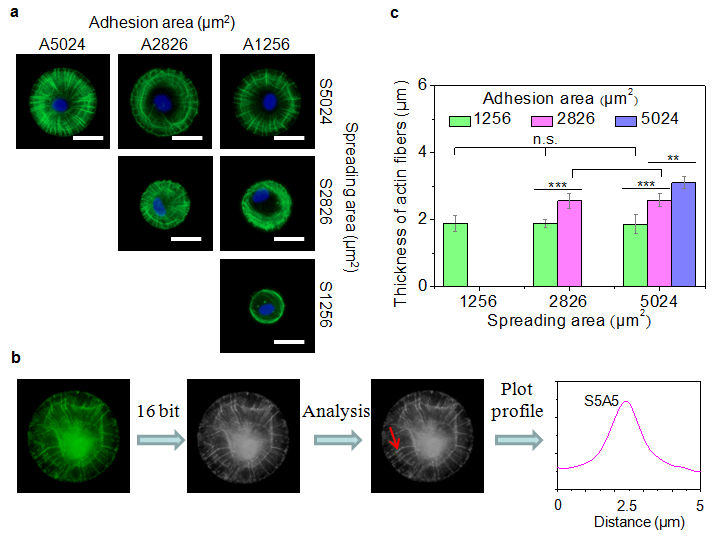
*

**Figure S4.** (a) Representative pictures of actin filaments (green). Nuclei: blue. Scale bar: 50 μm. (b) The analysis method to obtain the thickness of actinin fibers by ImageJ software. (c) The thickness of actin filaments on heterogeneous microarrays. The data present mean ± SD, n = 5, n.s., no significance; ***P*<0.01; ****P*<0.001.


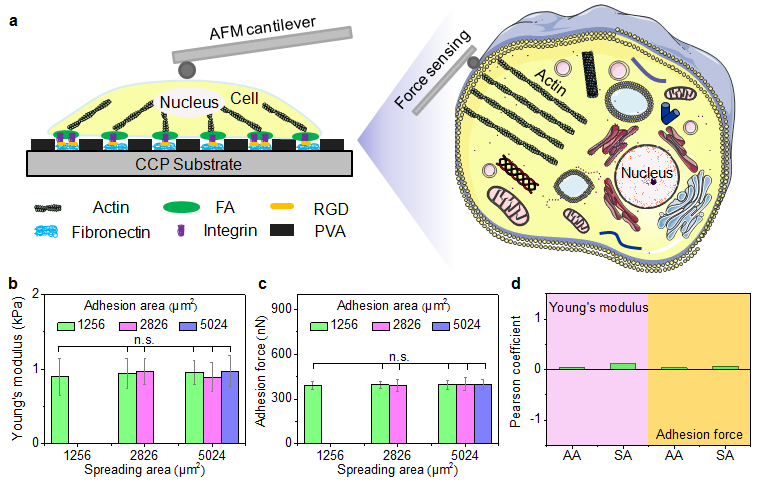


**Figure S5.** (a) Illustration of cell nanomechanical measurement by AFM on the microarrays. (b) Young’s modulus of the engineered hMSCs after disturbing actin filaments. (c) Adhesion force of the engineered hMSCs after disturbing actin filaments. The data present mean ± SD, n = 5, n.s., no significance. (d) Pearson coefficient analysis between cell adhesion behaviors and Young’s modulus or adhesion force after actin disturbance.


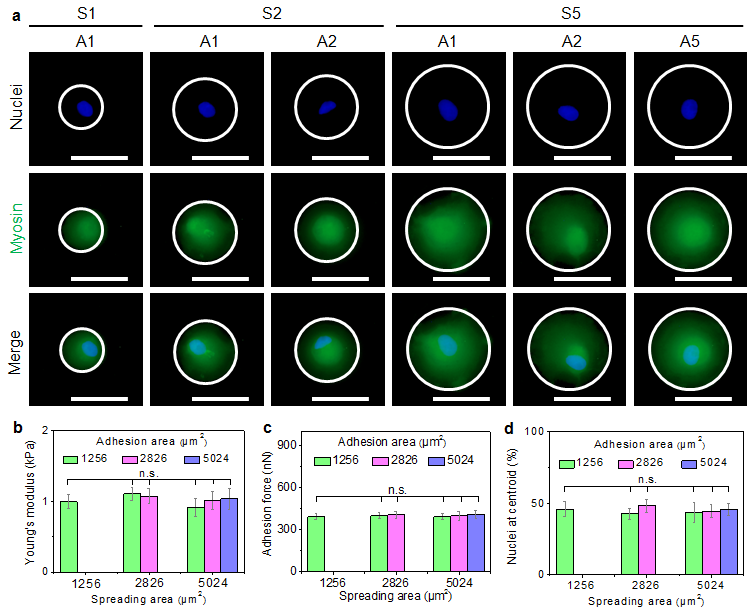


**Figure S6.** (a) Representative pictures of motor molecules. Myosin: green; nuclei: blue. The last row is merged pictures. Scale bar: 50 μm. The white circles present cell spreading areas on heterogeneous microarrays. (b) Young’s modulus after myosin disturbance. (c) Adhesion force after myosin disturbance. (d) Percentage of nuclei at centroid. The data present mean ± SD, n = 5, n.s., no significance.


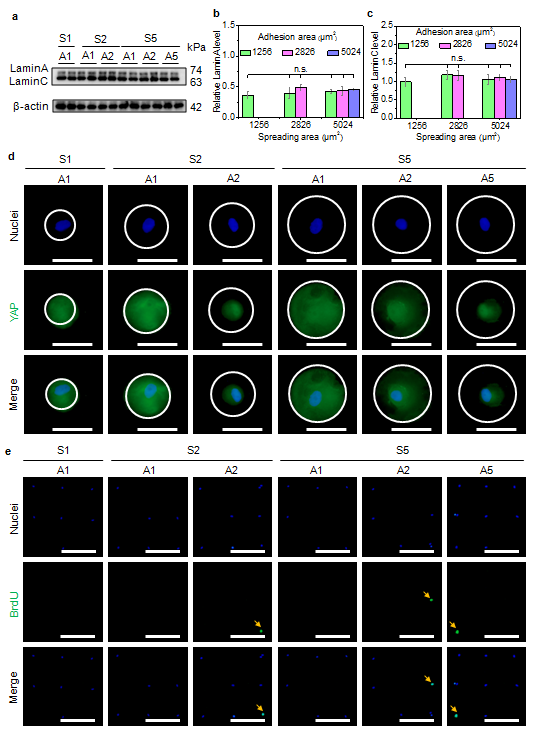


**Figure S7.** (a) LaminA/C measurement by WB analysis after actin disturbance. (b) Related LaminA expression level after actin disturbance. (c) Related LaminC expression level after actin disturbance. The data present mean ± SD, n = 4, n.s., no significance. (d) Representative pictures of YAP staining (green). Scale bar: 50 μm. The white circles present cell spreading areas on heterogeneous microarrays. (e) Representative pictures of BrdU staining (green). Nuclei: blue. Scale bar: 200 μm. The yellow arrows point out BrdU-positive nuclei.
